# Dorsomedial Striatal Activity Tracks Completion of Behavioral Sequences in Rats

**DOI:** 10.1523/ENEURO.0279-21.2021

**Published:** 2021-11-17

**Authors:** Youna Vandaele, David J. Ottenheimer, Patricia H. Janak

**Affiliations:** 1 Department of Psychological and Brain Sciences, Krieger School of Arts and Sciences, Johns Hopkins University, Baltimore, MD 21218; 2 The Solomon H. Snyder Department of Neuroscience, Johns Hopkins School of Medicine, Johns Hopkins University, Baltimore, MD 21205

**Keywords:** action sequence, dorsomedial striatum, goal-directed behavior, time processing

## Abstract

For proper execution of goal-directed behaviors, individuals require both a general representation of the goal and an ability to monitor their own progress toward that goal. Here, we examine how dorsomedial striatum (DMS), a region pivotal for forming associations among stimuli, actions, and outcomes, encodes the execution of goal-directed action sequences that require self-monitoring of behavior. We trained rats to complete a sequence of at least five consecutive lever presses (without visiting the reward port) to obtain a reward and recorded the activity of individual cells in DMS while rats performed the task. We found that the pattern of DMS activity gradually changed during the execution of the sequence, permitting accurate decoding of sequence progress from neural activity at a population level. Moreover, this sequence-related activity was blunted on trials where rats did not complete a sufficient number of presses. Overall, these data suggest a link between DMS activity and the execution of behavioral sequences that require monitoring of ongoing behavior.

## Significance Statement

Dorsomedial striatal activity was recorded during a task requiring rats to track progress in the execution of lever press sequences. Dorsomedial striatal activity evolved across the behavioral sequence with a ramp-like pattern of activity, permitting accurate decoding of sequence progress at the population level. Additionally, the magnitude of sequence-related activity was blunted on incomplete trials, suggesting that DMS activity may be critical for proper monitoring and execution of behavioral sequences. This study demonstrates that DMS neurons encode progress toward a goal during execution of action sequences when animals are required to track their own behavior for efficient performance.

## Introduction

Goal-directed behaviors depend on outcome expectation to guide the behavioral response (Dickinson, 1989; Dickinson and Balleine, 1994). However, goal-directed responses are rarely isolated actions. They often involve the execution of complex action sequences to attain the goal (Dezfouli and Balleine, 2013; Dezfouli et al., 2014), for instance, in the case of a predator hunting its prey. For optimal performance in this situation, individuals not only require a general representation of the goal of their actions (catching the prey) but also need to track their own progress toward that goal (approaching the prey).

The dorsomedial striatum (DMS) plays a pivotal role in forming associations among stimuli, actions and outcomes (Yin et al., 2005; Balleine et al., 2009; Balleine and O’Doherty, 2010; Corbit and Janak, 2010). Electrophysiological recording studies typically report that neurons in DMS are prominently modulated during execution of action sequences, in response to reward-predictive cues, and at the time of reward consumption (Barnes et al., 2005; Thorn et al., 2010; Ito and Doya, 2015; Jin and Costa, 2015; Rueda-Orozco and Robbe, 2015; London et al., 2018; Robbe, 2018; Vandaele et al., 2019). Interestingly, a growing number of studies also show a role for dorsal striatum in temporal processing. Specifically, the firing dynamics of striatal populations reliably encode time over tens of seconds in Pavlovian and instrumental tasks involving delays and timing behavior (Matell et al., 2003; Gouvêa et al., 2015; Mello et al., 2015; Bakhurin et al., 2017; Emmons et al., 2017). Furthermore, inactivation of the striatum significantly impairs interval timing (Gouvêa et al., 2015; Akhlaghpour et al., 2016; Emmons et al., 2017), a cognitive process that may contribute to tracking behavioral progress toward a goal during execution of action sequences.

Given this collection of evidence that DMS is involved in the execution of behavioral sequences and timing, we set out to characterize the activity of DMS neurons in a task requiring rats to monitor their own progress while performing lever press sequences. Specifically, rats had to complete a sequence of at least five consecutive lever presses without entering the reward port to obtain a reward. Thus, some sort of monitoring of action output would improve overall reward acquisition in this task. We found that DMS neuronal activity evolved across the behavioral sequence in a ramp-like pattern, permitting accurate decoding of sequence progress at the population level. Additionally, the magnitude of sequence-related activity was blunted on incomplete trials, suggesting that DMS neuronal activity may be critical for proper monitoring and execution of behavioral sequences.

## Materials and Methods

### Subjects

Long Evans rats (*N* = 9, 4 males, 5 females; Envigo) were used in this experiment and trained during the light cycle of the temperature (21°C) and light-controlled vivarium (12/12 h light/dark cycle, lights ON at 7 A.M.). Rats were individually housed and maintained under light food restriction (90% of free feeding weight, water *ad libitum*). This study was conducted in accordance with the recommendations of the *Guide for the Care and Use of Laboratory Animals* (Institute of Instrumental Training Laboratory Animal Resources, Commission of Life Sciences, National Research Council, 1996). The protocol was approved by the institutional animal care and use committee of Johns Hopkins University.

### Behavioral training

Rats were first trained to retrieve small aliquots of a solution of 20% sucrose (0.1 ml delivered over 3 s) during a single magazine training session (random interval 60 s for 30 min). Rats were then trained under a fixed ratio 1 schedule of reinforcement for three to five sessions (session limits: 1 h or 30 reward deliveries). After acquisition of instrumental responding, rats underwent surgeries and training in the fixed sequence 5 task began following the postoperative period of 5 d.

In this task, rats had to repress entering into the port before the completion of sequences of at least five consecutive lever presses to obtain a reward, whose delivery was not signaled. Premature port entries (before completion of the ratio) were penalized by resetting the ratio, and the preceding lever press sequence was considered as incomplete. Additional presses after completion of the ratio were without consequences and considered as part of the complete sequence. Rats were first trained with a fixed sequence length of two lever presses for a minimum of three sessions or until they earned 30 rewards. The response requirement was then increased to three lever presses for a minimum of two sessions (or until earning 30 rewards) before training in the final fixed sequence length 5 schedule (FS5) for 16–24 sessions. Sessions were limited to 30 min or 30 reward deliveries. We only analyzed neuronal activity during FS5 sessions after stabilization of performance in the current study (from the eighth FS5 session, for 9–17 sessions). The house-light, located on the ceiling of the operant chamber, housed within sound-attenuating boxes (Med Associates), remained illuminated during sessions.

### Surgeries and recording

Rats were implanted with unilateral arrays of eight electrodes (0.004’ insulated tungsten, with two silver ground wires) aimed at DMS with the following coordinates: +0.25 mm AP, ±2.3 mm ML, −4.6 mm DV. Surgeries were performed under isoflurane anesthesia (0.5–5%) with application of topical lidocaine for local analgesia and preoperative injections of antibiotic (cefazolin: 75 mg/kg) and analgesic (carprofen: 5 mg/kg). Rats were first accustomed for a few sessions under FR1 to tethering with the recording cable before training in the FS5 task. Cables connecting rats’ headsets to a commutator allow free movement throughout acquisition of single-unit activity with the multichannel acquisition processor (MAP) neural recording system (Plexon Inc). Electrode arrays were lowered at the end of every other session by 160-μm increments with a microdrive. To avoid duplicates, only units from one session were included for the analysis at any electrode location.

### Analysis of electrophysiological recordings

#### Spike sorting

The MAP neural recording system (Plexon Inc) was used to store and process amplified signals and timestamps of behavioral events. Analyses of interspike intervals distribution, auto-correlograms and cross-correlograms were conducted using Offline Sorter v3 and Neuro-Explorer 3.0 (Plexon Inc) to isolate individual unit offline. Timestamps and waveforms were exported from Neuro-Explorer 3.0 to MATLAB (The MathWorks) for further analysis. Analyses were restricted to units with well-defined waveforms and constant characteristics throughout the entire recording session.

### Waveform analysis

All the analyses in this study were restricted to neurons classified as putative medium spiny neurons (MSNs) according to waveform and firing rate properties. Units classified as putative interneurons were excluded. Putative fast spiking interneurons (FSIs) were defined by a firing rate higher than 20 Hz and narrow waveforms with half-valley width lower than 0.15 ms (*N* = 41; 3.6%). Units were classified as tonically active interneurons (TANs) when the firing rate was lower than 5 Hz, the half-valley width was higher than 0.45 ms, and the coefficient of variation of interspike intervals was lower than 1 (*N* = 17; 1.5%; Inokawa et al., 2010). Neurons not classified as interneurons but showing features intermediate to MSNs and interneurons were also excluded (range 12.5–20 Hz in firing rate and 0.4–0.45 ms in half-valley width; *N* = 80). As previously reported, population of putative-FSI and putative-TAN represented <5% and 1% of recorded units, respectively (Schmitzer-Torbert and Redish, 2008; Stalnaker et al., 2016; Martiros et al., 2018; Extended Data Fig. 2-1).

### Definition of task events and normalization of sequence related activity

Complete sequences were defined by sequences of at least five lever presses preceding a rewarded port entry whereas incomplete sequences comprised less than five lever presses and terminated with a premature port entry. Sequences longer than 20 s or followed by a latency to enter the port longer than 10 s were not considered. Neural firing rates during complete and incomplete lever press sequences were normalized according to the sequence duration. Specifically, the time to each spike in sequences was divided by the sequence duration, such that the first and last lever presses were considered as time 0 and 1, respectively; 100-bin histograms were generated for each sequence and frequency values were divided by the bin duration to estimate the firing rate. Similarly, activity during the port approach was normalized according to the port entry latency, such that the last lever press and the port entry were considered as time 0 and 1, respectively; 25-bins histograms were generated for each port approach and frequency values were divided by the bin duration to estimate the firing rate. The average bin width was 49.5 ± 0.4 ms for the lever press sequence (100 bins) and 41.5 ± 0. 3 ms for the port approach (25 bins). The 1-s periods preceding the first lever press and following the port entry were included in analysis and corresponded to the lever approach and reward consumption, respectively. Activity during these periods was computed using 25 time bins of 40 ms. Concatenated firing rate of individual neurons during lever approach, lever press sequence, port approach and reward consumption periods (consisting in a 175-bin vector) was smoothed (MATLAB function makedist, half-normal distribution, μ = 0, σ = 5) and *z* scored as follow: (Fi-Fmean)/Fsd. Fsd and Fmean represent the standard deviation and mean firing rate across the full behavioral sequence, and Fi is the firing rate at the ith bin of the behavioral sequence. Individual neurons were considered as excited or inhibited during lever pressing if the average *z* score from the first to the last lever press was positive or negative, respectively.

The same analyses were conducted using an event-centered approach (Extended Data Fig. 2-2). Firing rate was analyzed during 0.4-s time windows around each of the five lever press events and around the port entry (40-ms time bins, 20 bins per event). The lever press events were defined as follow: the first and second lever presses, one randomly selected intermediate lever press, the second to last lever press and the last lever press (Extended Data Fig. 2-2). Firing rate across the behavioral sequence (consisting in a 160-bin vector) was smoothed and *z* scored as described above.

### Principal component analysis (PCA)

A PCA (pca function in MATLAB) was conducted on the activity during the full behavioral sequence, including lever approach, lever presses, port approach and reward consumption periods. Spiking activity during lever presses was normalized according to the sequence duration, as described above. Similarly, activity during the port approach was normalized according to the port entry latency. This analysis was restricted to complete sequences and was conducted on a matrix of 1014 variables (number of DMS neurons included in the analysis) and 175 observations (number of time bins). The score values of the first two principal components (PCs) representing the most prevalent activity patterns among the neuronal population were analyzed.

### Decoding

A linear discriminant analysis (LDA) model (the fitcdiscr function in MATLAB) was trained on spike activity over relative time, achieved by delineating five equivalent consecutive intervals of the sequence to classify the position of each interval in the sequence. LDA models were trained on 95% of trials and used to classify the interval position in the remaining 5% of trials. To restrict the analysis to a matched number of trials, we combined across sessions and subjects neurons recorded during sessions with at least 26 complete sequences of duration shorter than 20 s and with a port entry latency shorter than 10 s. Subsequently, we restricted the analysis to 26 randomly selected trials. For ensemble decoding, we pooled together separately recorded units. We found the 26-fold cross-validated accuracy for models trained on the activity of randomly selected levels of 10, 50, 100, 500, and 900 units. We performed this analysis 50 times for each level. The same analysis was conducted with the interval positions shuffled to determine the accuracy expected from chance. Two-way ANOVAs were conducted to assess the effects of interval positions and ensemble size, on the decoding accuracy.

For single-unit decoding, we performed the analysis described above 26 times in a 26-fold cross-validation approach and averaged performance across all 26 repetitions to find that unit’s accuracy. To account for the variability in decoding accuracy resulting from random selection of trials, the analysis was repeated 20 times. To determine whether individual neurons predicted the position of a given interval above chance, we compared the decoding accuracy of each individual neuron for any time interval with the accuracy of the whole population of neurons after independently shuffling the firing activity of each unit across time intervals.

LDA models were also used to predict whether lever press sequences were complete or incomplete based on the activity during the approach of the lever (1 s before LP1, lever approach LA), after the first lever press (0.5 s post-LP1), before the last lever press (0.5 s pre-LLP), during the port approach or during the period of reward consumption. Every time periods were tested separately. Only neurons from sessions with at least 10 complete and 10 incomplete sequences were included in the analysis. The analysis was therefore conducted on the activity of 164 neurons across 20 randomly selected complete and incomplete trials (10 of each) with a 10-fold cross-validation approach. To account for the variability in decoding accuracy resulting from random selection of trials, the analysis was repeated 100 times.

For the analyses described above, neurons with extremely low firing rate (with null firing rate values in >75% of trials) were excluded to avoid errors from creating an LDA model on a dataset with too little variance. We assessed whether decoding accuracy significantly departed from chance (shuffled data) using permutation test.

LDA assumes that predictor variables are normally distributed. Thus, for each decoding analysis, we applied a box-cox normalization to predictor variables using the MATLAB function boxcox (Leontitsis A (2021) Box-Cox transformation. MATLAB Cent File Exch). The optimal λ for box-cox normalization was estimated with the MATLAB function boxcoxlm (Dror H (2021) Box-Cox power transformation for linear models. MATLAB Cent File Exch). We controlled for any bias in predictive accuracy by training LDA models on shuffled data to estimate the LDA accuracy expected from chance. Finally, decoding analyses were also conducted using random forest classifiers (Breiman, 2001; Liaw and Wiener, 2002), not subjected to normality assumptions (Extended Data Figs. 3-2, 5-1). Similar to LDA analyses, the random forest classifiers were trained on spike activity over relative time, achieved by delineating three, five, or seven equivalent consecutive intervals of the sequence to classify the position of each interval in the sequence (function classRF_train). Random forest classifiers were trained on 95% of trials and used to classify the interval position in the remaining 5% of trials with the function classRF_predict (Liaw and Wiener, 2002). Using the same approach, random forest classifiers were also trained on the activity during the approach of the lever, after the first lever press, before the last lever press, during the port approach or during the period of reward consumption, to predict whether lever press sequences were complete or incomplete. The ntree and mtry parameters were set to default [500 and floor(sqrt(size(X,2)), respectively].

### Statistical analysis

Data following a normal distribution were subjected to repeated measures analysis of variance. The Kruskal–Wallis test was used when normality assumption was violated. Mean *z* scores were compared across complete and incomplete sequences using two-tailed Student’s *t* tests. All analyses were conducted on MATLAB (MathWorks).

### Histology

Electrode sites were labeled by passing a DC current through each electrode, under deep anesthesia with pentobarbital. All rats were perfused intracardially with 1 m PBS followed by 4% paraformaldehyde. Brains were extracted, postfixed in 4% paraformaldehyde for 4–24 h, and transferred in 20% sucrose for >48 h for cryo-protection. To verify electrode placement, the brains were sectioned at 50 μm on a cryostat and slices were stained with cresyl violet and analyzed using light microscopy.

## Results

### Rats monitor their performance during execution of fixed-length lever press sequences

**Figure 1.**
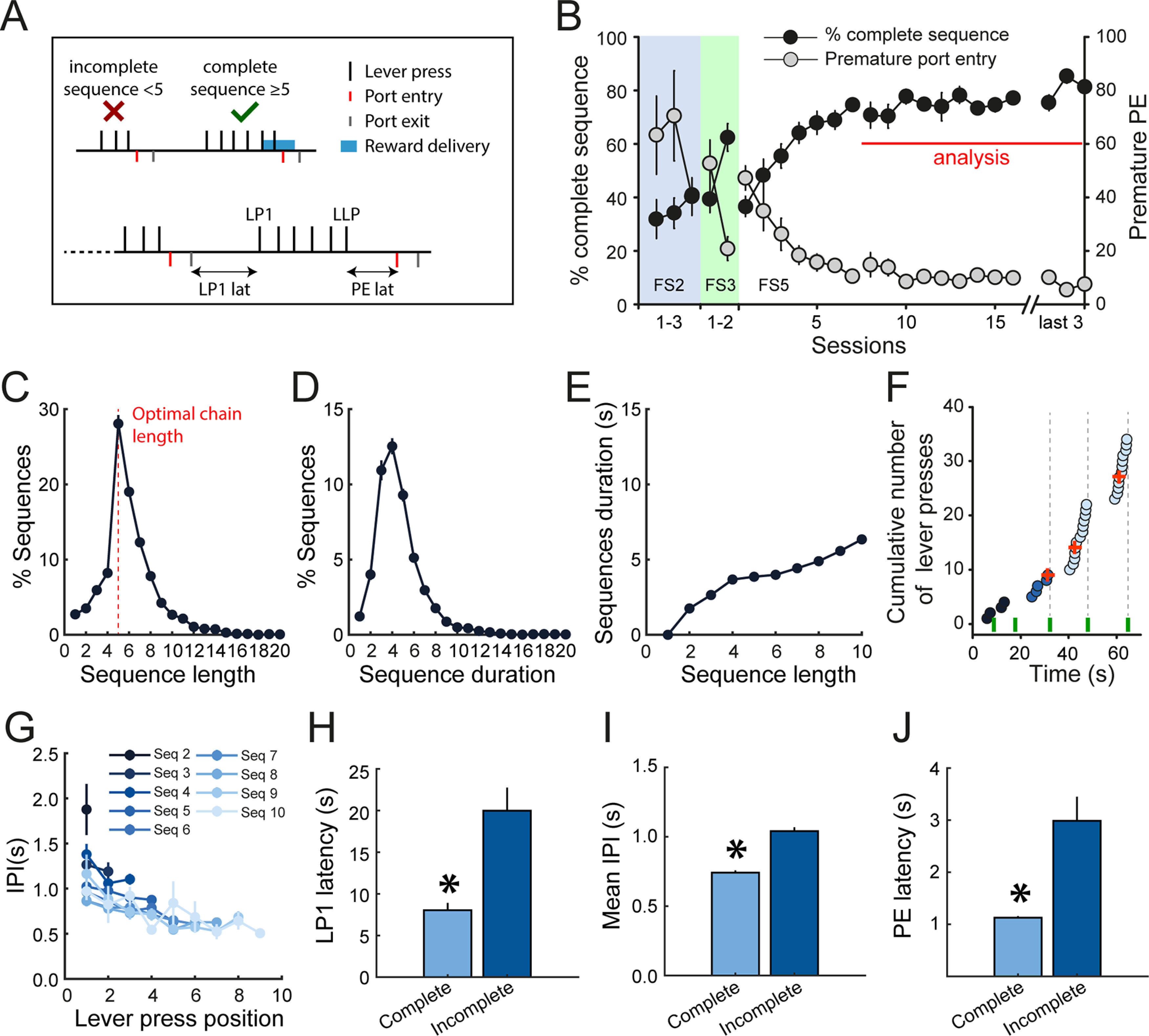
Rats monitor their performance during execution of fixed-length lever press sequences. ***A***, Diagram of the FS5 task. LP1: first lever press. LLP: last lever press. PE: port entry. ***B***, Mean percentage of completed sequences and number of premature port entries across training sessions. ***C***, ***D***, Mean distribution of sequence length (***C***) and duration (***D***). ***E***, Mean sequence duration as a function of sequence length. ***F***, Microstructure of behavior during execution of sequences in a representative rat, at the start of the session. Circles: lever presses. Green ticks: port entries. Red crosses: reward deliveries. ***G***, Mean inter-press intervals (IPIs) as a function of sequence length and across lever press position. ***H–J***, Mean LP1 latency (***H***), IPI (***I***), and PE latency (***J***) for complete and incomplete sequences; *p < 0.001. ***C–E***, ***G–J*** represent the mean (±SEM) of 118 FS5 sessions (Extended Data Fig. 1-1).

Rats were trained to complete sequences of consecutive lever presses without checking the reward port to obtain a reward. Premature visits of the reward port were penalized by resetting the response requirement (Fig. 1*A*). The acquisition of the sequence was gradual; after initial instrumental training under a continuous reinforcement schedule, the subjects (*N* = 9) were trained with a sequence length of two and then three lever presses before training at the final sequence length of five lever presses (FS5) for 16–24 sessions (Materials and Methods). Completion of the full lever press sequence without checking the port increased over time, as indicated by the rise in percentage of complete sequences, reaching an asymptote at 80% across the last three recording sessions (main effect of sessions: *F*_(20,140)_ = 13.96, *p* < 0.0001; Fig. 1*B*), and by a decrease in the number of premature port entries (main effect of sessions: *F*_(20,140)_ = 10.17, *p* < 0.0001; Fig. 1*B*). We analyzed behavior and DMS neuronal spiking activity after stabilization of performance from the eighth FS5 session (Fig. 1*B*).

10.1523/ENEURO.0279-21.2021.f1-1 Extended Data Figure 1-1 Analysis of behavior combined across sessions and averaged across rats. ***A***, ***B***, Mean distribution of sequence length (***A***) and duration (***B***). Gray plots represent individual rats. ***C***, Mean sequence duration as a function of sequence length. ***D***, Mean IPIs as a function of sequence length and across lever press position. ***E–G***, Mean LP1 latency (***E***), IPI (***F***), and PE latency (***G***) as a function of sequence length. Data represent the mean (±SEM). This figure refers to Figure 1. Download Figure 1-1, TIF file.

Analysis of rats’ lever pressing revealed that rats were monitoring their reward-seeking behavior to perform the task optimally (Fig. 1*C*,*D*). Rats most frequently made five lever presses, indicating that they had learned the response contingency and titrated their behavior to the optimal chain length (Fig. 1*C*). Rats may have also achieved high performance in the task by estimating the time elapsed from the first lever press. Analysis of the distribution of sequence durations reveals a peak at 4 s (Fig. 1*D*), which corresponds to the average time for the completion of sequences four to six presses in length (Fig. 1*E*).

We also examined whether there were any changes in pressing behavior within individual sequences that could indicate that the subjects were actively monitoring sequence progress (Fig. 1*F*,*G*). Indeed, rats were faster to press the lever as they approached the end of the sequence (Fig. 1*F*), illustrated by decreases in the mean interpress intervals (IPIs) as subjects progressed through the sequence (sequences 4–9: *F* values > 4.5, *p* values < 0.05; Fig. 1*G*). Overall, rats initiated, executed, and terminated complete sequences more quickly than incomplete sequences, suggesting that they were less motivated on trials for which they did not complete the lever press requirement (latency to the first press: *F*_(1,116)_ = 21.2, *p* < 0.001; mean IPI: *F*_(1,116)_ = 210.9, *p* < 0.001; PE latency: *F*_(1,116)_ = 16.44, *p* < 0.001; Fig. 1*H–J*). Analyses of individual rats showed similar results (Extended Data Fig. 1-1).

### DMS activity is characterized by a ramp across lever presses followed by a switch in activity at the approach of the port

**Figure 2.**
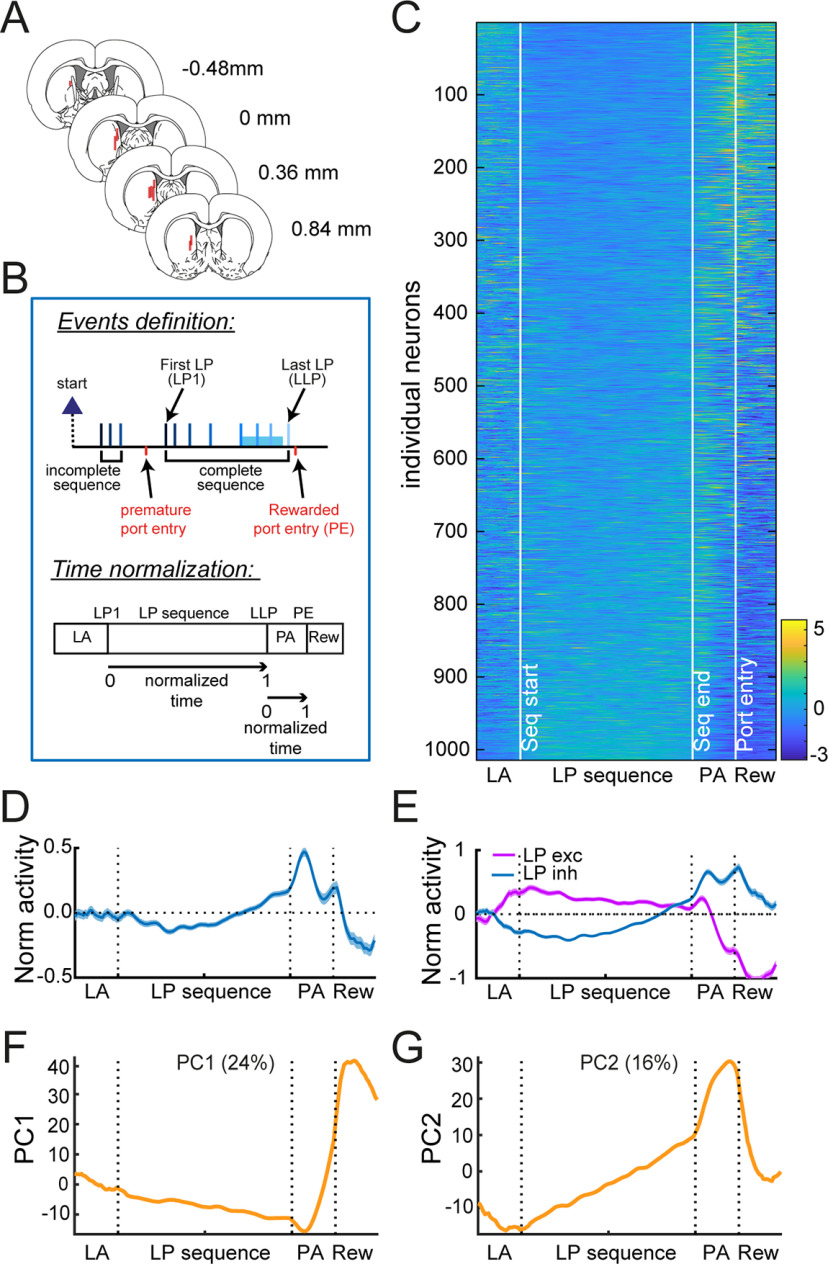
DMS neural activity is characterized by a ramp across lever presses followed by a switch in activity at the approach of the port. ***A***, Electrode placements. ***B***, Diagram of task events and normalization of spiking activity according to sequence duration and port entry latency. LA: lever approach; PA: port approach; Rew: reward. ***C***, ***D***, Heatmap (***C***) and average *z* score (±SEM; ***D***) of MSNs. ***E***, Average *z* score (±SEM) of MSNs with relative excitation or inhibition during lever presses. ***F***, ***G***, Eigenvector values of PC1 (***F***) and PC2 (***G***). The % variance explained by each component is indicated (Extended Data Figs. 2-1, 2-2).

To characterize the activity of putative MSNs (*N* = 1014, 88% of recorded units; Extended Data Fig. 2-1) in DMS (Fig. 2*A*) during the execution of lever-press sequences, we normalized the time elapsed from the first (LP1) to the last (LLP) lever press (Fig. 2*B*; Materials and Methods). In this way, we could measure relative changes in firing across the sequence. Because we observed that the latency to enter the port depended on the sequence length (Kruskal–Wallis test: χ^2^ = 333, *p* < 0.0001), the same normalization approach was employed between the last lever press and the port entry (port approach period, PA).

10.1523/ENEURO.0279-21.2021.f2-1 Extended Data Figure 2-1 Identification of MSNs. ***A***, 3D scatter plot of coefficient of variation of interspike intervals, half-valley width and firing rate allowing separation of TANs, MSNs, and FSIs. ***B***, Example of the average waveform of putative-TAN, putative-MSN, and putative-FSI. ***C***, Example of autocorrelograms of putative-TAN, putative-MSN, and putative-FSI. ***D***, Mean (±SEM) coefficient of variation of interspike intervals in putative-FSI, putative-MSN, and putative-TAN. Main effect of neuron type: *p* < 0.0001; different letters indicate statistical differences. ***E***, Mean (±SEM) cumulative density function of interspike intervals in putative-FSI, putative-MSN, and putative-TAN. As previously shown (Inokawa et al., 2010), putative-TAN exhibited longer and more regular interspike intervals compared to putative-FSI and putative-MSN (***D***, ***E***). This figure refers to Figure 2. Download Figure 2-1, TIF file.

10.1523/ENEURO.0279-21.2021.f2-2 Extended Data Figure 2-2 Characterization of DMS activity with an event-centered approach. ***A***, Diagram of task events and analysis. ***B***, ***C***, Heatmap (***B***) and average *z* score (±SEM; ***C***) of MSNs. ***D***, Average *z* score (±SEM) of MSNs excited or inhibited during lever presses. This figure refers to Figure 2. Download Figure 2-2, TIF file.

We first analyzed MSN activity during complete sequences (greater or equal to five lever presses). The normalized activity of individual MSNs is depicted in Figure 2*C*. While the average normalized activity of this population decreased during the first lever presses, rose toward the end of the sequence, peaked after the last lever press, and dropped during the port approach (Fig. 2*D*), the activity of individual units is clearly more variable. When neurons were separated based on the sign of their mean *z* score during lever presses (relative excitation or relative inhibition), we observed a switch in activity during the port approach (Fig. 2*E*), with a relative decrease in firing for neurons excited during lever presses and a relative increase in firing for neurons inhibited during lever presses. We note that a comparable pattern of activity was observed when firing rate was analyzed in 40-ms time bins around the lever press and port entry events (−0.25- to 0.25-s perievents; Extended Data Fig. 2-2). We also characterized the population activity using a PCA. This analysis revealed that, across the population, there tended to be ramps of neural activity across the sequence and sharp transitions in firing during port approach and reward acquisition (Fig. 2*F*,*G*).

### Progress in the lever press sequence is encoded in DMS activity pattern

**Figure 3.**
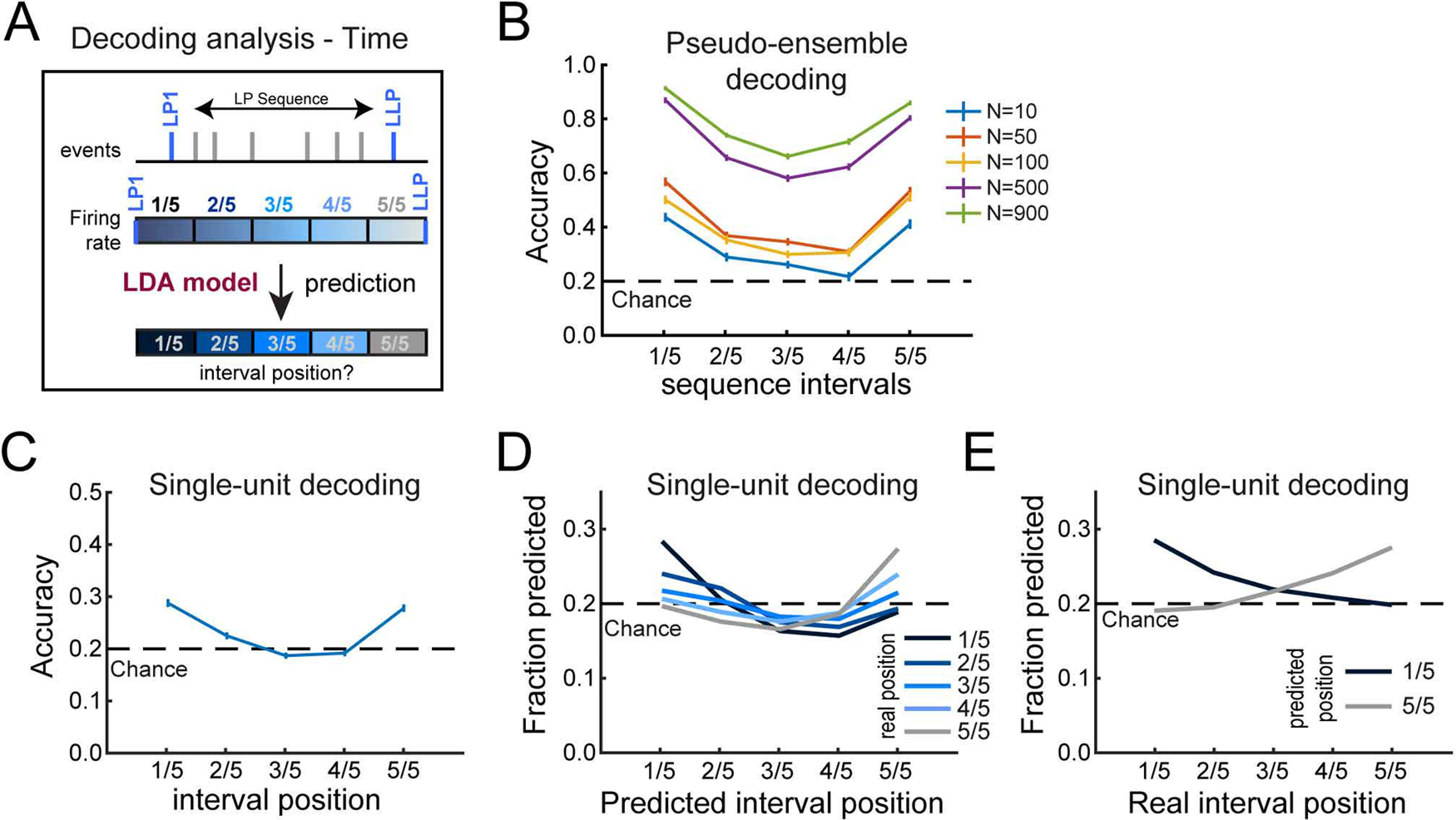
Progress in the lever press sequence is encoded in the DMS neural activity pattern. ***A***, Diagram of the decoding analysis. ***B***, Mean decoding accuracy across time intervals and as a function of pseudo-ensemble size. ***C***, Mean single-unit decoding accuracy across time intervals. ***D***, Fraction of predicted interval position as a function of real interval position. ***E***, Fraction of intervals predicted as the first (1/5) and the last (5/5) interval as a function of real interval position (Extended Data Figs. 3-1, 3-2, 3-3).

The gradual shift in neuronal activity during sequence execution suggested that the animal’s progress in the execution of the lever press sequence could be read out from DMS neuronal firing. To test this hypothesis, we trained LDA models on the firing rates of individual DMS neurons across five equivalently sized, consecutive intervals of the sequence on a subset of trials. We used these models to classify the sequence position of the intervals for held-out, test trials (Fig. 3*A*). For this analysis, we pooled neurons recorded from sessions comprising at least 26 complete sequences of duration shorter than 20 s and with a latency to retrieve the reward shorter than 10 s (*N* = 903). We first conducted this analysis on randomly selected pseudo-ensembles of neurons. For ensemble sizes of *N* = 50 and above, LDA models accurately predicted the time positions of all held-out sequence intervals (permutation tests: *p* values < 0.0001; Fig. 3*B*). The accuracy increased with the ensemble size (reaching 65–91% for *N* = 900 neurons), and differed as a function of interval position (main effect of ensemble size, *F*_(4,1249)_ = 1096.7 *p* < 0.0001; main effect of interval position, *F*_(4,1249)_ = 331.3, *p* < 0.0001; interaction *F*_(16,1249)_ = 3.0, *p* < 0.0001). Interestingly, the accuracy was higher at the first and last intervals compared with intermediate intervals (*post hoc p* values < 0.0001). Similar results were found when LDA models were trained on activity across fewer or greater number of consecutive time intervals (Extended Data Fig. 3-1). These results were also replicated with a random forest classifier (Extended Data Fig. 3-2).

10.1523/ENEURO.0279-21.2021.f3-1 Extended Data Figure 3-1 DMS activity pattern tracks progress across time intervals of the behavioral sequence. ***A–C***, Mean single-unit decoding accuracy (±SEM) across time intervals of sequences subdivided in three (***A***), five (***B***), and seven (***C***) equivalently sized consecutive intervals. ***D–F***, Mean decoding accuracy (±SEM) as a function of pseudo-ensemble size and across time intervals of sequences subdivided in three (***D***), five (***E***), and seven (***F***) equivalently sized consecutive intervals. This figure refers to Figure 3. Download Figure 3-1, TIF file.

10.1523/ENEURO.0279-21.2021.f3-2 Extended Data Figure 3-2 Decoding analysis with a random forest classifier. ***A–C***, Mean decoding accuracy (±SEM) as a function of pseudo-ensemble size and across time intervals of sequences subdivided in three (***A***), five (***B***), and seven (***C***) equivalently sized consecutive intervals. This figure refers to Figure 3. Download Figure 3-2, TIF file.

**Figure 4:**
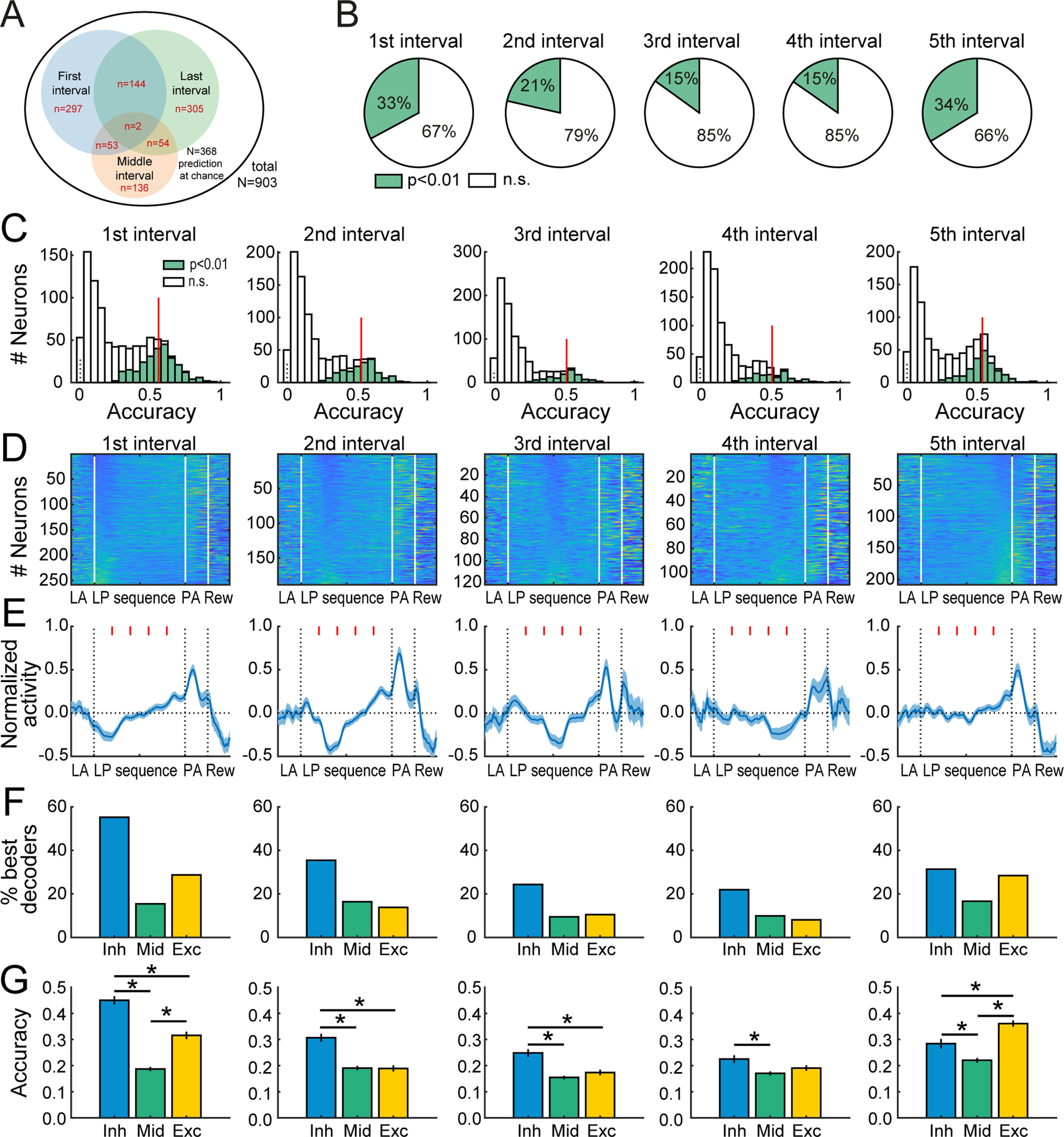
Stronger decoding at beginning and end of sequence by individual neurons. ***A***, Venn diagram of neurons predicting the position of the first, third, and last interval above chance. ***B***, Proportion of individual neurons that predict the position of each interval above chance, beginning at the first lever press and ending at the last lever press. ***C***, Distribution of decoding accuracy of individual neurons that best predict each of five time intervals. ***D***, ***E***, Heatmaps (***D***) and average *z* score (±SEM; ***E***) of neurons that best predict the position of an interval above chance, for each interval. Red ticks in ***E*** mark the limit of consecutive intervals. Activity during the lever approach, port approach, and reward consumption are shown but not included in the decoding analysis. ***F***, ***G***, Proportion (***F***) and mean (±SEM) predictive accuracy (***G***) of neurons that best predict an interval, in the most inhibited (Inh), moderately modulated (mid), and the most excited neurons (Exc). Neurons were classified as Inh, Mid, or Exc if they ranked below, between, or above the lower and upper quartile of the *z* score distribution, respectively (Extended Data Fig. 3-3). * *p* < 0.05.

10.1523/ENEURO.0279-21.2021.f3-3 Extended Data Figure 3-3 Progress in the lever press sequence is encoded in DMS activity pattern. ***A***, Diagram of the decoding analysis with an event-centered approach. ***B***, Mean decoding accuracy (±SEM) across lever presses and as a function of pseudo-ensemble size. ***C***, Mean single-unit decoding accuracy (±SEM) across lever presses. ***D***, Fraction of predicted lever press position as a function of real lever press position. ***E***, Fraction of lever press predicted as the first and the last press as a function of real lever press position. ***F***, Proportion of individual neurons that best predicted the position of each lever press event above chance. ***G***, Distribution of decoding accuracy of individual neurons that best predicted each of the lever press events. ***H***, ***I***, Heatmaps (***H***) and average *z* score (±SEM; ***I***) of neurons that best predicted the position of a lever press event above chance, for each lever press. This figure refers to Figures 3, 4. Download Figure 3-3, TIF file.

With evidence that sequence progress is encoded at the population level, we next investigated whether individual neurons were sufficient to decode sequence position. On average, individual neuron activity could also predict above chance the position of the first, second and last sequence intervals (permutation tests; first: *p* < 0.0001; second: *p* < 0.05; last: *p* < 0.0001; Fig. 3*C*), although the overall accuracy was poor. Interestingly, there was a pattern to the misclassification errors. Overall, time intervals were more likely classified as the first interval the closer they were to the sequence initiation and, correspondingly, were more likely classified as the last interval the closer they were to the sequence termination (Fig. 3*D*). Accordingly, the fraction of neurons from which the first interval is predicted decreased (first vs last interval McNemar χ^2^ = 580.3, *p* < 0.0001) whereas the fraction of neurons from which the last interval position is predicted increased (McNemar χ^2^ = 533.2, *p* < 0.0001), as subjects progressed through the sequence (Fig. 3*E*). Similar results were found by training LDA models on normalized spike activity around each individual lever press to decode the lever press position in the sequence (Extended Data Fig. 3-3). These results are consistent with the ramping pattern of activity observed in DMS neurons along the behavioral sequence (Fig. 2) and indicate that activity in DMS gradually progresses in a reliable pattern as the sequence is completed.

We next analyzed the activity of individual neurons that predicted the position of a given interval above chance by comparing single-unit decoding accuracy to the accuracy of the whole population of neurons after their activity was shuffled (Fig. 4; Materials and Methods). A majority of individual neurons accurately predicted at least one specific time interval (*p* < 0.01 for at least one interval in 59% of neurons, 535 out of 903; Fig. 4*A*), with larger proportions of good decoders for the first and last intervals (first interval *N* = 297, 33%; fifth interval: *N* = 305, 34%; Fig. 4*B*). Among these, numerous neurons concurrently decoded the location of both the first and last intervals (*N* = 144), whereas overlaps with the middle interval (third interval) were more limited (first and third interval: *N* = 53; third and fifth interval: *N* = 54; Fig. 4*A*). Since some neurons decoded several intervals’ location, we grouped neurons by their best predicted position (i.e., each neuron only represented once in this analysis), and found an effect of interval on their decoding accuracy (mean for each interval, first: 0.56; second: 0.52; third: 0.51; fourth: 0.52; fifth: 0.53; main effect of interval: *F*_(4,883)_ = 5.64, *p* < 0.001; Fig. 4*C*). Examination of the heatmaps (Fig. 4*D*) and the average activity pattern of best decoders (Fig. 4*E*) illustrates that neurons decoding the first and last interval positions resembled the prominent activity patterns across the population in Figure 2, that is, these neurons’ activity tended to ramp across the sequence, permitting reliable decoding of the beginning and end of the sequence. Interestingly, with the exception of the last interval, we systematically observed a transient relative inhibition during the interval best predicted by the neurons (Fig. 4*E*). Accordingly, neurons that were the most inhibited during a given interval (in the lower quartile of the mean *z* score distribution) were more likely to predict the position of that interval above chance (χ^2^ > 21, *p* values < 0.0001; Fig. 4*F*) and showed greater predictive accuracy (Kruskal–Wallis test *p* values < 0.05; Fig. 4*G*). For the last interval, the most inhibited and excited neurons contributed equally to the predictive accuracy (Fig. 4*F*,*G*). Similar activity patterns were observed when the LDA models were trained on the firing rates around each individual lever press (Extended Data Fig. 3-3). This analysis demonstrates that, at the level of individual neurons, there is robust encoding of aspects of the sequence, characterized by a ramp toward termination of the sequence, and single-unit inhibition during the predicted intervals. Yet, none of the neurons encoded every interval of the sequence, suggesting that strong population encoding of behavioral progress toward a goal (Fig. 3*B*) emerges from single units individually encoding fewer time intervals but collectively encoding the full behavioral sequence at a population level.

### Attenuated activity pattern during incomplete sequences

**Figure 5.**
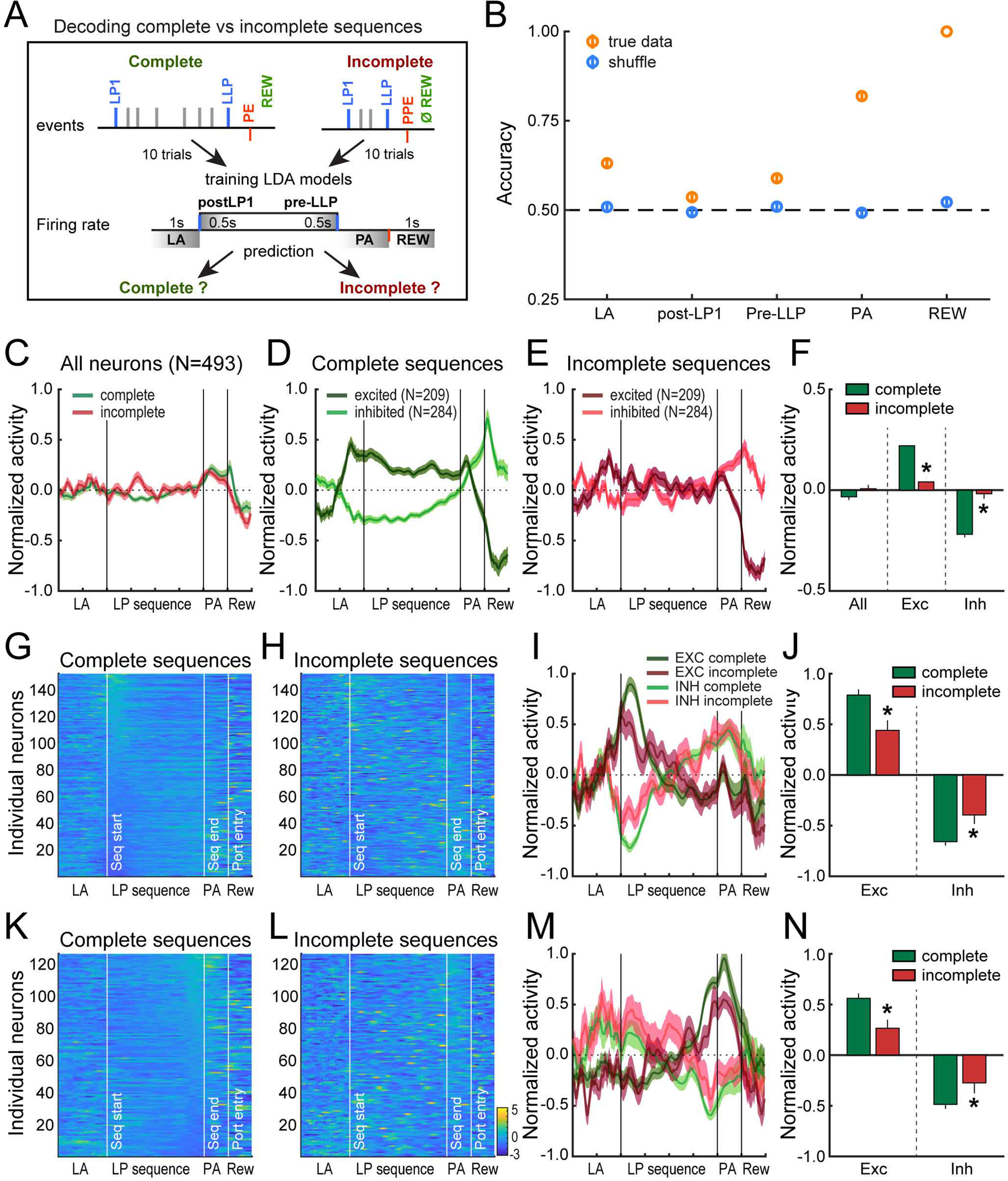
Attenuated neural activity patterns during incomplete sequences. ***A***, Diagram of the decoding analysis. ***B***, Decoding accuracy in true and shuffled conditions across time events in the behavioral sequence. Independent LDA analyses were conducted separately for each time event. ***C***, Average *z* score (±SEM) of MSN for complete and incomplete sequences. ***D***, ***E***, Mean *z* score (±SEM) of MSN for complete (***D***) and incomplete (***E***) sequences, separated based on their mean activity during lever presses in complete sequences. ***F***, Mean *z* score (±SEM) during lever presses for complete versus incomplete sequences for all MSN and for excited or inhibited MSN; *p < 0.0001. ***G***, ***H***, Heatmaps of first interval decoders during complete (***G***) and incomplete sequences (***H***). ***I***, ***J***, Average *z* score (±SEM) of first interval decoders, separated based on their modulation during the first interval in complete versus incomplete sequences. ***J***, Average *z* score (±SEM) of excited and inhibited first interval decoders during the first interval; *p < 0.01. ***K–N***, Same as ***G–J*** for the last interval decoders; *p < 0.05 (Extended Data Fig. 5-1).

The sequence decoding analysis demonstrates that DMS neuronal firing tracks rats’ progress in the execution of complete lever press sequences. We next sought to determine whether DMS neuronal activity differs when rats fail to execute a complete sequence, and, if so, when that difference might emerge. We trained LDA models on normalized spike activity during specific time points across complete and incomplete sequences to classify held-out trials as complete or incomplete (Fig. 5*A*). For this analysis, we pooled neurons from sessions comprising at least 10 complete and 10 incomplete sequences (*N* = 164). We made no assumption on whether DMS activity could differentiate complete versus incomplete sequences from neural activity before, after, or during performance of the lever presses themselves. Thus, we separately assessed the decoding accuracy from independent analyses of the activity at the beginning and end of the sequence (0–0.5 s post-LP1 and −0.5–0 s pre-LLP), but also outside of the sequence during the lever approach (−1–0 s pre-LP1), the port approach (from LLP to PE), and the time of expected reward consumption (0–1 s post-PE; Fig. 5*A*). We assessed whether the decoding accuracy significantly departed from chance by comparing it with the accuracy after shuffling complete and incomplete sequences.

10.1523/ENEURO.0279-21.2021.f5-1 Extended Data Figure 5-1 Decoding of complete versus incomplete sequences with a random forest classifier. Decoding accuracy in true and shuffled conditions across time events in the behavioral sequence. Independent random forest analyses were conducted separately for each time event. This figure refers to Figure 5. Download Figure 5-1, TIF file.

LDA models accurately distinguished complete from incomplete sequences at each time point of the sequence, but there were significant differences across events (*F*_(4,499)_ = 370.0, *p* < 0.0001). Specifically, mean accuracy for assignment to complete or incomplete sequences was significantly above chance for all events (permutation tests: *p* values < 0.0001, except that *p* < 0.05 for the beginning of the sequence; Fig. 5*B*). Complete and incomplete sequences could be dissociated from normalized DMS activity before the first lever press when rats approached the lever to initiate the sequence (mean accuracy = 0.63 ± 0.01), suggesting that a difference in neural state preceded the initiation of complete versus incomplete sequences and was maintained throughout their execution and termination. This difference in neural state parallels well the difference in motivational state illustrated by longer first lever press latency, mean IPI and port entry latency in incomplete sequences relative to complete sequences, showing that rats were less engaged in the task on trials when they made a premature port entry (Fig. 1*H–J*). We found higher decoding accuracy at the port approach (mean accuracy = 0.82 ± 0.01; Fig. 5*B*) and, as would be expected, perfect accuracy at the time of reward feedback (mean accuracy = 1). The sensory cues (sight, smell, sound of reward delivery) that signal reward delivery in complete trials, and its absence in incomplete trials, might contribute to the relatively high decoding accuracy at the port approach time point. Surprisingly, the accuracy to classify sequences as complete or incomplete was the lowest at the beginning and termination of the sequence (mean accuracy, post-LP1: 0.54 ± 0.01, pre-LLP: 0.59 ± 0.01; Fig. 5*B*), suggesting that task accuracy is better predicted from the neural activity outside of the sequence. Similar results were found with a random forest classifier (Extended Data Fig. 5-1).

Since we were able to dissociate complete and incomplete sequences based on DMS neuronal firing, we next asked how DMS activity differed between these two types of trials. We selected MSNs from sessions comprising at least five complete and incomplete sequences (*N* = 493). Comparison of the average *z* score along the lever press sequence revealed no obvious difference between complete and incomplete sequences (*t* = −1.85, *p* = 0.065; Fig. 5*C*,*F*). However, when neurons were separated based on the sign of their mean *z* score during lever presses in complete sequences (excitation *N* = 209 or inhibition *N* = 284), we observed stronger modulations in activity during execution of compete sequences (Fig. 5*D*) compared with incomplete sequences (Fig. 5*E*), with larger *z* scores during proper execution of the sequence (excitation *t* = 5.7, *p* < 0.0001; inhibition *t* = −7.91, *p* < 0.0001; Fig. 5*F*). In contrast, during incomplete sequences, we observed a flatter pattern during lever approach and lever presses and a lower peak during the port approach in inhibited neurons (Fig. 5*E*). These results suggest that the dynamic sequence-related activity pattern of DMS neurons is attenuated when rats are less engaged in the task during incomplete sequences.

We further examined the activity of neurons previously identified as encoding the first and last intervals of lever press sequences (Fig. 4) on complete and incomplete sequences (first interval predictors *N* = 153; last interval predictors *N* = 127 in these sessions; Fig. 5*G*,*H*,*K*,*L*). When neurons encoding the first time interval of complete sequences were separated according to the direction of their modulation during the first interval, we observed a larger peak in activity for complete sequences relative to incomplete sequences (first interval excitation: *t* = 3.72, *p* < 0.001; inhibition: *t* = −3.2, *p* < 0.01; Fig. 5*I–J*). When neurons encoding the last interval of complete sequences were separated according to their modulation at the end of the sequence, we also observed a larger peak in complete sequences compared with incomplete sequences, specifically in neurons increasing their firing rate (last interval excitation: *t* = 3.14, *p* < 0.01; inhibition: *t* = −2.05, *p* < 0.05; Fig. 5*M*,*N*). These results show that disengagement from the task during incomplete sequences is accompanied by dampened activity patterns in DMS neurons encoding behavioral progress at the initiation and termination of the lever press sequence.

## Discussion

Here, we sought to determine whether on-line tracking of the execution of behavioral sequences could be observed in DMS neuronal spiking activity. Using a task in which subjects were penalized for checking for reward before sequence completion, we found that rats learned to track their own behavior and titrated their responding to the optimal response chain length. Efficient performance in this task was associated with a specific neural activity pattern in the DMS, characterized by a ramp across lever presses followed by a switch in activity as rats approached the port to retrieve the reward. It was possible to predict from this neural activity pattern (1) progress in the execution of the sequence and (2) whether a sequence was complete or not. Together, these results suggest that neural activity in the DMS tracks progress in the execution of action sequences, and may allow rats to estimate when to check the port to retrieve the expected reward.

Rats carefully monitored execution of lever press sequences in the FS5 task, most often making five consecutive responses, corresponding to the optimal chain length for the maximization of reinforcement rate. This pattern of responding is consistent with other tasks requiring execution of action chains with a minimal number of lever presses for the reward to be delivered (Evenden, 1998a; van Haaren and van Hest, 1990; Rivalan et al., 2007; Vandaele et al., 2018). In absence of feedback cues indicating the correct completion of the sequence, rats had to rely on a representation of their progress in the sequence to determine when to check the port, either by estimating the number of lever presses emitted or by estimating the time elapsed from the first lever press. Interestingly, we observed an acceleration of lever press responses as rats progressed in the execution of the sequence, a “scalloping pattern” also found in fixed interval schedules involving timing behavior across delays (Dews, 1978; Wearden and Lejeune, 2006). This response pattern is thought to represent an increase in expectation as rats get closer to the expected time of reward delivery and suggests that efficient performance in the FS5 task may involve time processing.

The ramping pattern of neural activity found here is also reported in studies investigating striatal encoding of time during Pavlovian and instrumental tasks involving delays and timing behavior (Matell et al., 2003; Donnelly et al., 2015; Gouvêa et al., 2015; Mello et al., 2015; Emmons et al., 2017). In the present study, DMS neural activity was characterized by a smooth ramp across lever presses followed by a switch in activity as rats approached the port to retrieve the reward. Upward and downward ramps across lever presses could be an expectation signal that grows as rats progress closer to the reward (Hikosaka et al., 1989; Apicella et al., 1992; Howe et al., 2013), whereas the switch in activity at the port approach could represent a reorganization of striatal ensembles as rats terminate the lever press sequence to select the port entry action (Mink, 1996; Graybiel and Grafton, 2015). The ramps reported here could have resulted from the time normalization process we used, leading to a misalignment of lever press events combined with an increase in response rate toward the end of the sequence (Fig. 1). However, a similar ramping pattern was observed when we used an event-centered approach, ensuring the times of each lever press were aligned across trials (Extended Data Fig. 2-2). Although press-related peaks were observed with this approach, the peaks were higher later in the sequence, suggesting that action-related activity cannot account alone for the ramping pattern. This result suggests that DMS spiking activity integrates information about time and actions, as previously suggested (Mello et al., 2015; Emmons et al., 2017). Our results are however inconsistent with another study reporting action-related activity but no ramping pattern in the dorsal striatum during execution of lever press sequences (Ma et al., 2014b). Differences in task requirements may explain this discrepancy. While our task required rats to estimate their own progress in the sequence to reach the port at the right time, rats in the study of Ma and colleagues had to learn the correct sequential order of lever press responses but were not required to estimate the time elapsed or the number of lever presses emitted. This suggests that striatal ramping pattern during an action sequence is only observed when animals are actively tracking relative time or progress in its execution. Future lesion or inactivation studies are needed to directly demonstrate that disrupting DMS activity impairs sequence tracking in the FS5 task.

Previous findings indicate that ramping activity patterns in the striatum allow time estimation and that these ramps are scalable across multiple delays (Gouvêa et al., 2015; Mello et al., 2015; Bakhurin et al., 2017; Emmons et al., 2017). In agreement with these studies, we could predict progress through sequence execution from DMS neural activity with high accuracy for larger pseudo-ensembles of neurons. The higher decoding accuracy at the start and end of the sequence is striking and suggests stronger striatal encoding of the beginning and end of the sequence compared with its intermediate parts, perhaps because the first lever press “resets” an internal clock and triggers the ramp onset whereas activity at the end of the sequence reaches a threshold for the selection of the next port entry response (Matell and Meck, 2000; Narayanan, 2016). Interestingly, several studies have shown the emergence of neuronal excitations in the dorsal striatum at the borders of behavioral sequences, as individual actions are chunked into behavioral units across sequence learning (Jog et al., 1999; Jin and Costa, 2010; Jin et al., 2014; Smith and Graybiel, 2016; Martiros et al., 2018). Although the proposed notions of task-bracketing activity or start/stop-related activity remains a matter of debate (Robbe, 2018; Sales-carbonell et al., 2018; Vandaele et al., 2019), the stronger decoding accuracy at the beginning and end of the sequence may suggest a striatal encoding of sequence initiation and termination. While changes in spiking activity were observed at the termination of the sequence during the port approach, it is notable that the approach of the lever at sequence self-initiation was not characterized by phasic excitation or inhibition. More research is thus needed to directly relate our results to the presence of discrete start/stop signals in the dorsal striatum. In addition, in the current study, we collapsed neurons from different recording sessions to create pseudo-ensembles. Future studies should perform similar analyses on subsets of simultaneously recorded neurons. Although the decoding accuracy was high for the full lever press sequence in these large pseudo-ensembles of neurons, at the level of individual neurons, it was only possible to predict the position of one or two time intervals with a moderate accuracy. Our findings therefore suggest that at a population level, DMS neurons collectively track progress in the lever press sequence by integrating single-unit encoding of fewer time intervals.

Complete and incomplete sequences could be distinguished based on DMS spiking activity at several phases of the behavioral sequence. As expected, incomplete sequences were perfectly predicted from DMS activity after the port entry, when rats realized that the reward was not delivered. The decoding accuracy significantly departed from chance during execution of the sequence and reached 80% during the port approach, when reward expectation was the highest. However, we cannot exclude that sensory cues gradually predicted reward availability in complete trials, and its absence in incomplete trials, as rats approached the reward port. Paradoxically, while the beginning and end of the sequence could be predicted from neurons’ activity with a great accuracy, it was difficult to predict from these time points whether a sequence would be completed or not. Indeed, the mean decoding accuracy was only 0.54 and 0.59 after the first and before the last lever press, respectively. These results suggest that spiking activity did not substantially differ at the beginning and end of complete versus incomplete sequences and may constitute an indirect indication that ramping pattern of striatal activity instead scales with the sequence length. It is however worth noting that the decoding analysis was conducted on a limited number of trials (10 of each) and neurons (*N* = 164) because of the low number of incomplete sequences. This limit in neuron and trial number could result in a sampling bias with the exclusion of sessions with fewer incomplete sequences and could have contributed to the overall lower predictive accuracy in this analysis. Additional studies at earlier stages of training, when rats still make a high number of incomplete sequences, would thus be desirable, and as well, could reveal how striatal activity during complete versus incomplete sequences changes in step with behavior, as rats learn to monitor their own behavior.

Surprisingly, we also found significant decoding accuracy in the classification of complete versus incomplete sequence when analyzing spiking activity during the approach of the lever. In other words, it was possible to predict whether or not a sequence would be completed before it was initiated. This result indicates that neural activity differs between complete and incomplete sequences before sequence execution, which may parallel a difference in motivational state. Indeed, longer sequences were initiated, executed and terminated faster than short sequences, suggesting transient changes in motivation within the course of the session, rats being less motivated or less engaged in the task during shorter, incomplete sequences. This task disengagement was associated with attenuated activity patterns during incomplete sequences compared with complete sequences. Furthermore, neurons predicting the location of the first and last intervals were also less modulated at these times during incomplete sequences. This dampened activity pattern during incomplete sequence may constitute a neural marker of spontaneous failure to track execution of the sequence. However, since complete and incomplete sequences could be dissociated from activity at the lever approach and before initiation of the lever press sequence, this hypothesis is insufficient. Instead, the emission of a premature port entry could result from incorrect planning and motor impulsivity (Evenden, 1998a,b,c; Dalley et al., 2011; Dalley and Robbins, 2017). However, unlike incomplete sequences, impulsive actions are associated with faster response latencies than actions involving planning (Dalley et al., 2011; Dalley and Robbins, 2017), and impulsive actions in the five-choice serial reaction time task are not associated with attenuated striatal activity patterns (Donnelly et al., 2015). Therefore, we suggest that the attenuated activity pattern in DMS, in which there is a lower modulation of firing rate during execution and at sequence boundaries, could represent a lower motivational state of the animal, resulting in premature cessation of the sequence.

To conclude, we have shown that DMS neurons encode progress toward a goal during execution of action sequences when animals are required to track their own behavior for efficient performance. This striatal region receives numerous inputs from cortical areas, notably the prefrontal cortex (McGeorge and Faull, 1989; Hart et al., 2018). Ramping patterns of activity have been found in prefrontal brain regions and are proposed to play a role in top-down control of time processing in the dorsal striatum (Narayanan and Laubach, 2009; Kim et al., 2013; Ma et al., 2014a,b; Parker et al., 2014; Xu et al., 2014; Donnelly et al., 2015; Emmons et al., 2017). In addition, a ramping pattern of dopamine release emerges in the striatum as rats move toward distant goals (Howe et al., 2013) or during execution of lever press sequences (Wassum et al., 2012; Collins et al., 2016). This tonic ramp in dopamine signaling observed as subjects traverse real or virtual distance is proposed to reflect reward expectation (Howe et al., 2013) or the instantaneous reward prediction error (Kim et al., 2020) and may serve to support ongoing motivation to respond through a task. The importance of dopaminergic systems on timing behavior and motivational control is well-reflected by the severe impairments observed in patients suffering from Parkinson’s disease (Parker et al., 2013). Further research is needed to determine how the DMS integrates time estimates from prefrontal regions and motivational signals from dopamine circuits in tracking progress toward a goal during execution of action sequences.

## Synthesis

Reviewing Editor: Nathalie Ginovart, University of Geneva Decisions are customarily a result of the Reviewing Editor and the peer reviewers coming together and discussing their recommendations until a consensus is reached. When revisions are invited, a fact-based synthesis statement explaining their decision and outlining what is needed to prepare a revision will be listed below. The following reviewer(s) agreed to reveal their identity: Christian Lüscher, Mark Laubach.

Upon discussion, the reviewers expressed interest in your manuscript and agreed on the significance of the work as a novel contribution to the field. However, they also raised a number of issues that will need to be addressed in a revision. You will find a copy of these comments below.

Reviewer 1 :

In the present study, the authors examined how DMS activity encodes and tracks the progress of goal-directed behavioral sequences. The authors found that the pattern of DMS activity is characterized by a ramp across lever presses which switched at the approach of the port. By using LDA model, authors found DMS activity also changes during the progress of the sequences, and show a different pattern during complete or incomplete sequences.

The authors have designed an behavioral protocol required animals to monitor their own goal-directed behavior progress, the data is sufficiently analyzed and the manuscript is well written. I have some comments and suggestions to improve the manuscript and I have listed them below:

Major comments:

1. Only one experiment is conducted, with a set of different analysis. The main problem is it lacks a causal evidence that manipulating DMS impairs behavior sequencing tracking. While is may be sufficient for a first publication, the authors should discuss possible experiments that could be carried out in the future.

2. In the analysis of DMS activity, only sessions with at least 10 complete and 10 incomplete sequences are included. Would this lead to a sampling problem of excluding the good sessions with less incomplete sequences?

3. From figure 4D it seems like the neuron could predict each interval above chance level are those been activated/inactivated during this interval. Can you do the reverse, that find all neurons being activated/inactivated during each interval, and see how well they could predict the position?

Minor comments:

1. The y-label is normalized activity in Fig 2D-E and Fig4E, but z-score in fig 5. Need to be consistent if they are calculated in a same way

2. In calculating the normalized activity, the Fmean is obtained from the mean firing rate across the full behavioral sequence, which does not reflect the real baseline of non-activity period. Is it possible to use the mean firing rate of a period when animal is not performing any behaviour relevant to the task as Fmean?

3. Line 94: “Because we observed that the latency to enter the port depended on the sequence length (Fig. 1J)” Fig 1J showed a shorter latency to port entry in complete trails (with reward delivery) than incomplete trials (with no reward delivery), although completed trials are longer than imcomplete trials, it’s not appropriate to conclude the latency to enter the port depend on the sequence length without controlling the reward delivery.

Reviewer 2 :

This manuscript reports novel results on how neurons in the dorsomedial striatum of rats track sequential actions in a behavioral task that is reminiscent of classic studies using what was called the “fixed consecutive number” design. (Prior studies using this design were not cited, as far as I could tell, and the authors should review this issue in revising their manuscript.) The data analysis is generally well carried out but several issues were apparent to me that I feel should be addressed.

First, the classification of neurons did not address inter-spike interval statistics, and this could impact the classification of TANs. Classic studies (Kimura) showed that the coefficient of variation for inter-spike intervals is a good measure to discriminate between bursty spiny type neurons and tonically active neurons. This measure corresponds well to classic slice physiology for TANs in primates and rodents. Also, spiny type cells tend to fire in bursts (more on this below regarding normalization). The authors should include a summary of inter-spike interval and burst statistics to support their classification of cell types. As they used NEx for the analyses, the can easily do these analysis with routines that are available in that application.

Second, the bursty nature of spiny type cells raises issues for normalization using Z scores. What is the meaning of a negative score for a cell that mostly fires in bursts? The analyses should be done using an alternative form of normalization that maintains the positive nature of spiking. The Matlab neural network toolbox has routines for this purpose (normc), and if this toolbox is not available, there are freely available versions of the routines on the internet. This issue matters for LDA, which has a number of assumptions about the distributions of the predictor variables, including that they are normally distributed.

Third, my second point leads to this third issue. Is LDA a good choice for decoding? More recent classifiers, such as Random Forest and gradient boosting algorithms, are free of some of the limiting assumptions of LDA. The authors should show that they can obtain the same results with at least one other classifier. They should also evaluate if their collapsing across data sets is justified by analyzing ensembles of simultaneously recorded neurons. Their analysis only makes sense if striatal neurons have independent variability. If the trial-by-trial variations in firing rate are redundant over neurons, then their analysis overemphasizes the decoding results. If variability is synergistic (cells explicitly vary on different trials), then their approach underestimates the decoding.

Fourth, does bin size matter for the classifiers and PCA? Given the bursty nature of spiny cell firing, results should vary with bin size. A bin size of 40 ms was used. Do the results depend on this choice? Please evaluate all results using a range of bin sizes, perhaps using a grid search approach.
