# Supplementary figures and images for: Dorsomedial Striatal Activity Tracks Completion of Behavioral Sequences in Rats
Source: eNeuro. 2021 Nov 17;8(6):ENEURO.0279-21.2021. doi: 10.1523/ENEURO.0279-21.2021 (PMC8607909; doi:10.1523/ENEURO.0279-21.2021)

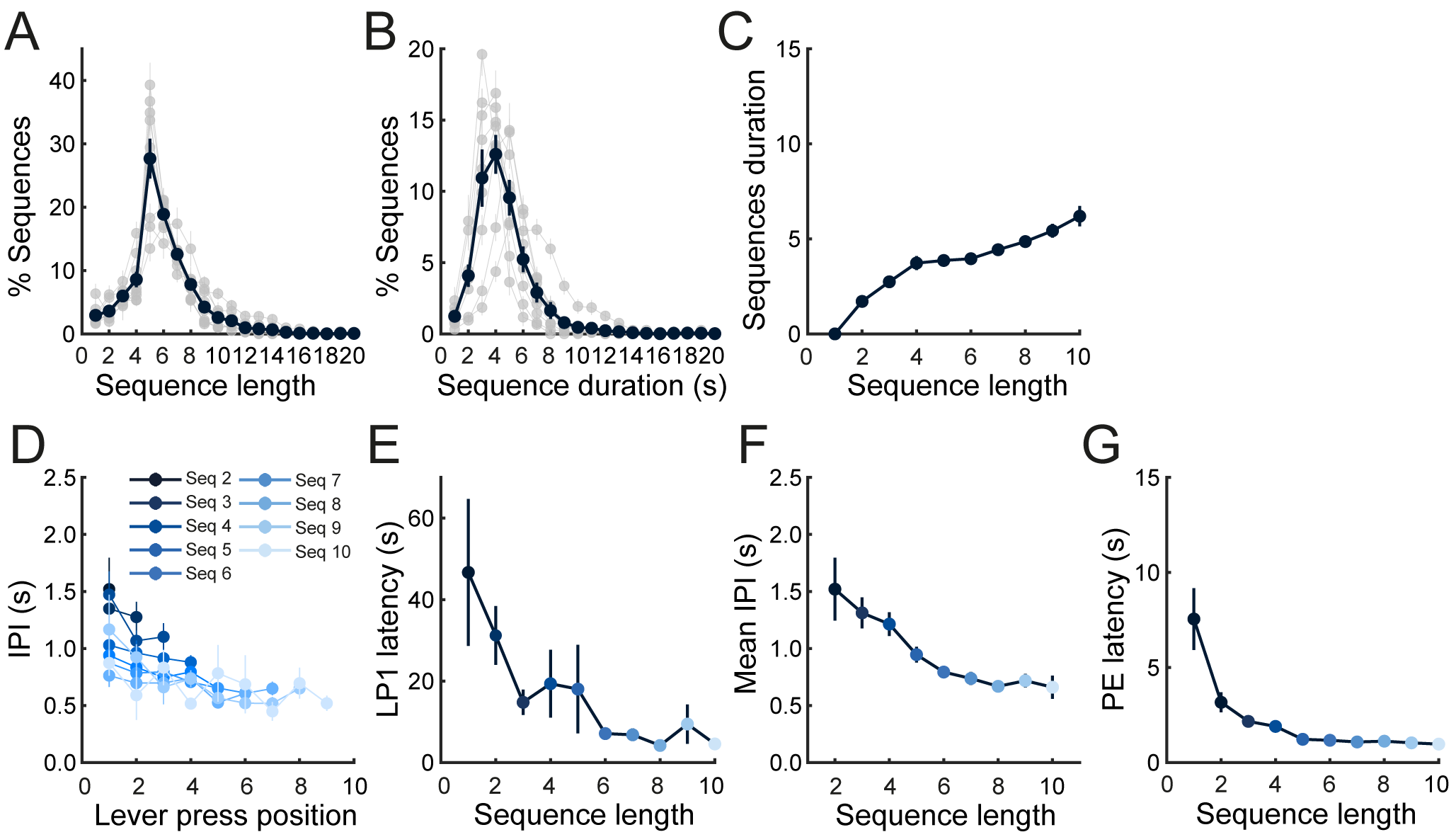

Supplement: Extended Data Figure 1-1 — Analysis of behavior combined across sessions and averaged across rats. A, B, Mean distribution of sequence length (A) and duration (B). Gray plots represent individual rats. C, Mean sequence duration as a function of sequence length. D, Mean IPIs as a function of sequence length and across lever press position. E–G, Mean LP1 latency (E), IPI (F), and PE latency (G) as a function of sequence length. Data represent the mean (±SEM). This figure refers to Figure 1. Download Figure 1-1, TIF file. [file enu-eN-NWR-0279-21-s02.tif]

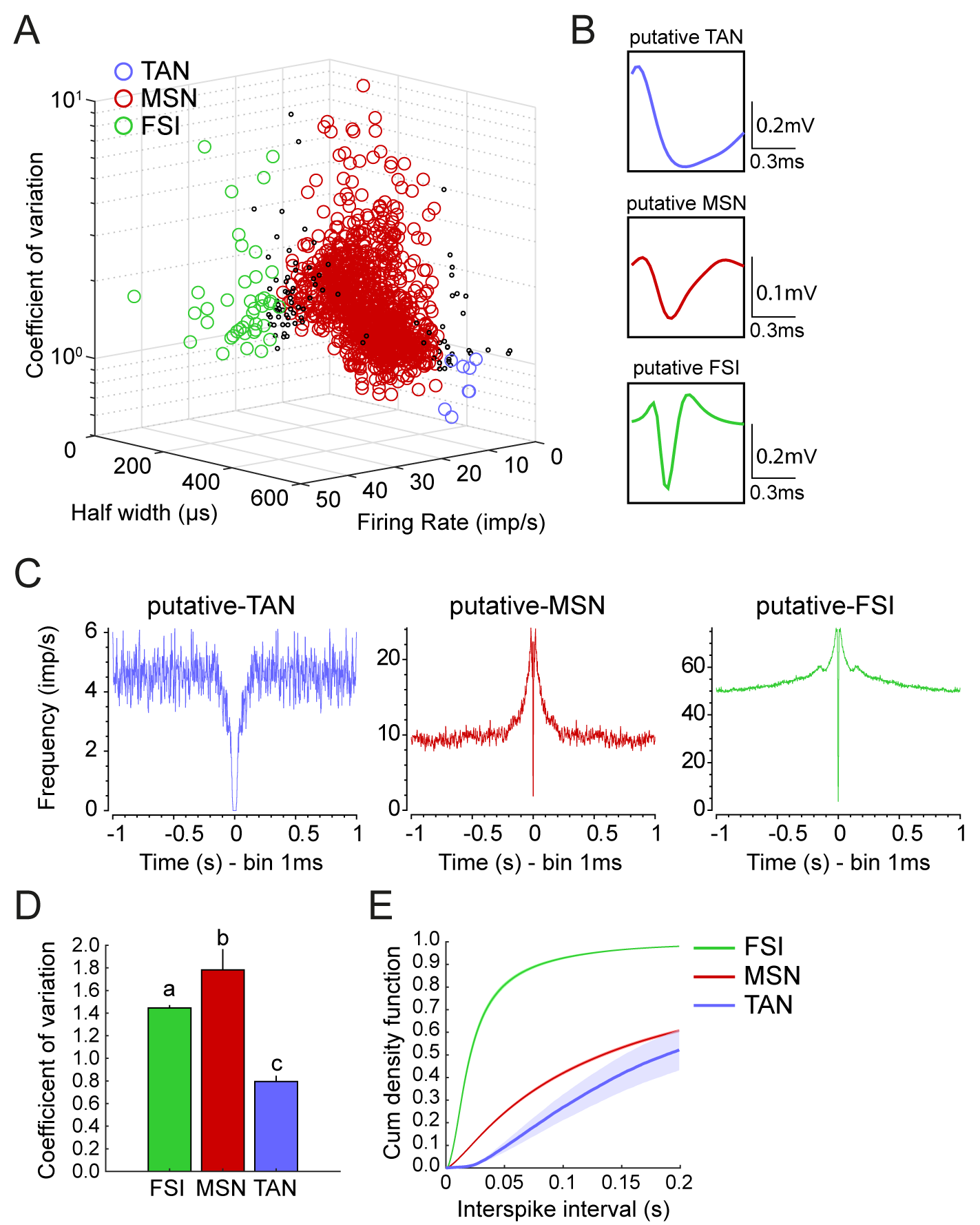

Supplement: Extended Data Figure 2-1 — Identification of MSNs. A, 3D scatter plot of coefficient of variation of interspike intervals, half-valley width and firing rate allowing separation of TANs, MSNs, and FSIs. B, Example of the average waveform of putative-TAN, putative-MSN, and putative-FSI. C, Example of autocorrelograms of putative-TAN, putative-MSN, and putative-FSI. D, Mean (±SEM) coefficient of variation of interspike intervals in putative-FSI, putative-MSN, and putative-TAN. Main effect of neuron type: p < 0.0001; different letters indicate statistical differences. E, Mean (±SEM) cumulative density function of interspike intervals in putative-FSI, putative-MSN, and putative-TAN. As previously shown (Inokawa et al., 2010), putative-TAN exhibited longer and more regular interspike intervals compared to putative-FSI and putative-MSN (D, E). This figure refers to Figure 2. Download Figure 2-1, TIF file. [file enu-eN-NWR-0279-21-s03.tif]

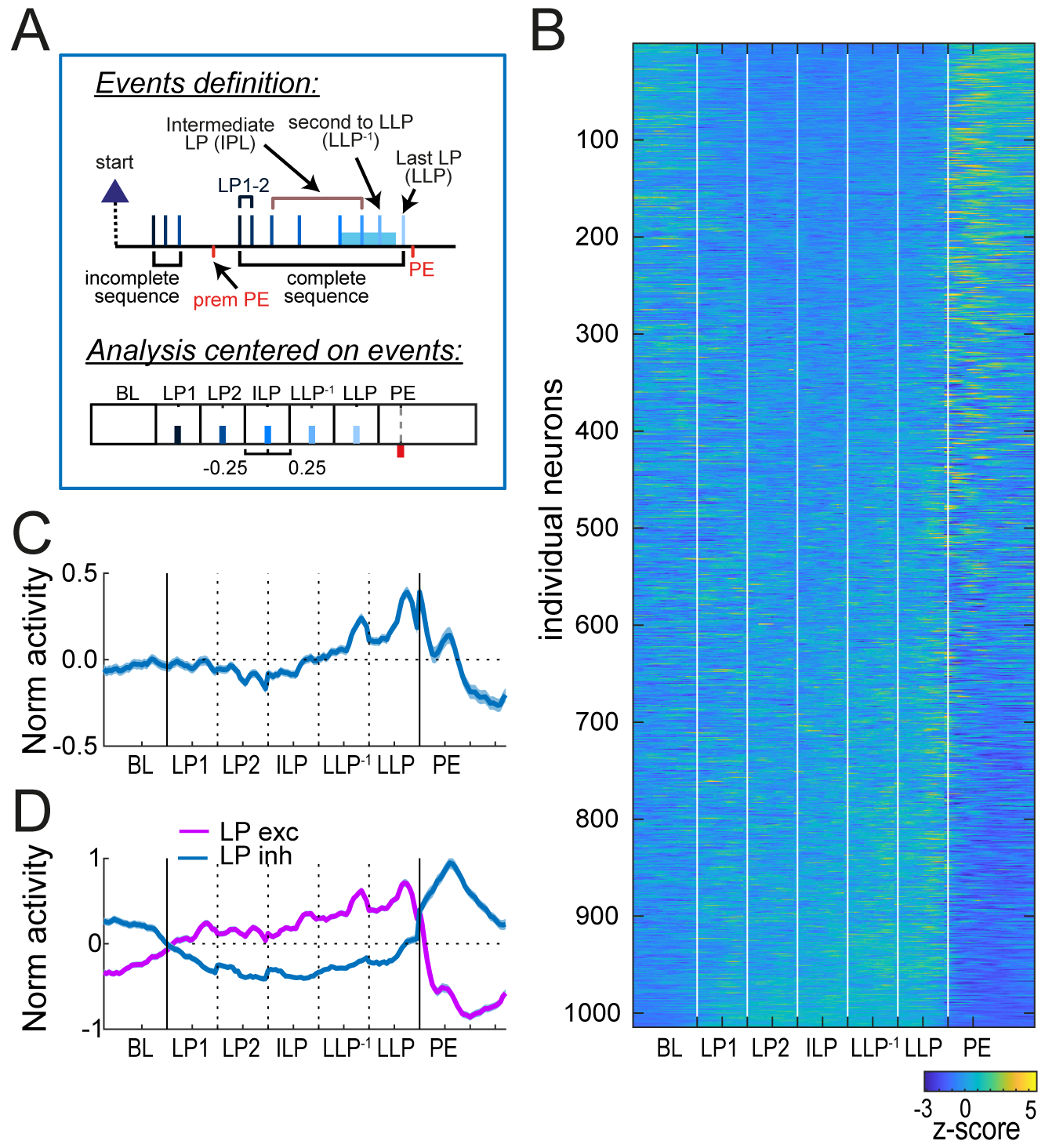

Supplement: Extended Data Figure 2-2 — Characterization of DMS activity with an event-centered approach. A, Diagram of task events and analysis. B, C, Heatmap (B) and average z score (±SEM; C) of MSNs. D, Average z score (±SEM) of MSNs excited or inhibited during lever presses. This figure refers to Figure 2. Download Figure 2-2, TIF file. [file enu-eN-NWR-0279-21-s04.tif]

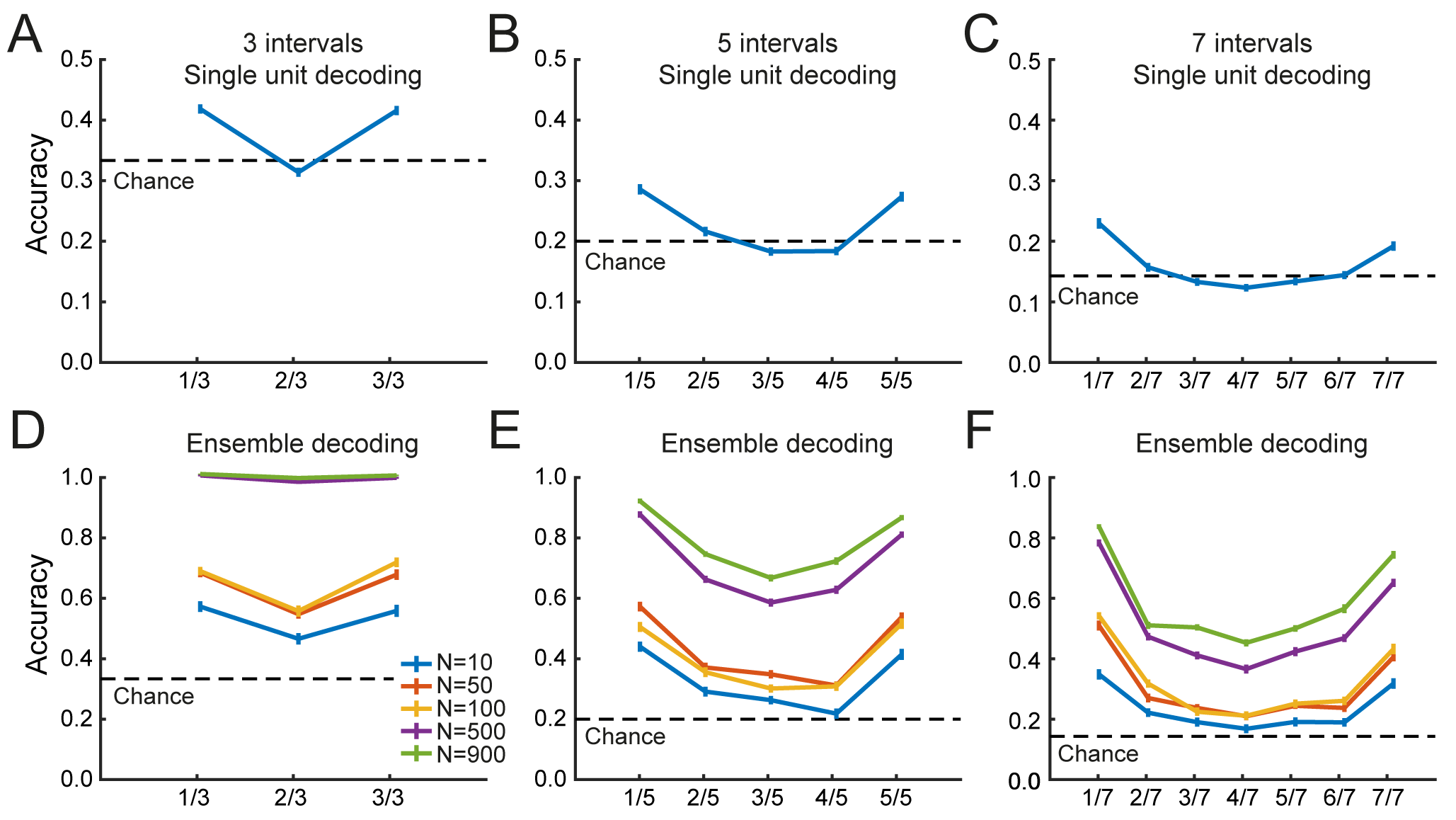

Supplement: Extended Data Figure 3-1 — DMS activity pattern tracks progress across time intervals of the behavioral sequence. A–C, Mean single-unit decoding accuracy (±SEM) across time intervals of sequences subdivided in three (A), five (B), and seven (C) equivalently sized consecutive intervals. D–F, Mean decoding accuracy (±SEM) as a function of pseudo-ensemble size and across time intervals of sequences subdivided in three (D), five (E), and seven (F) equivalently sized consecutive intervals. This figure refers to Figure 3. Download Figure 3-1, TIF file. [file enu-eN-NWR-0279-21-s05.tif]

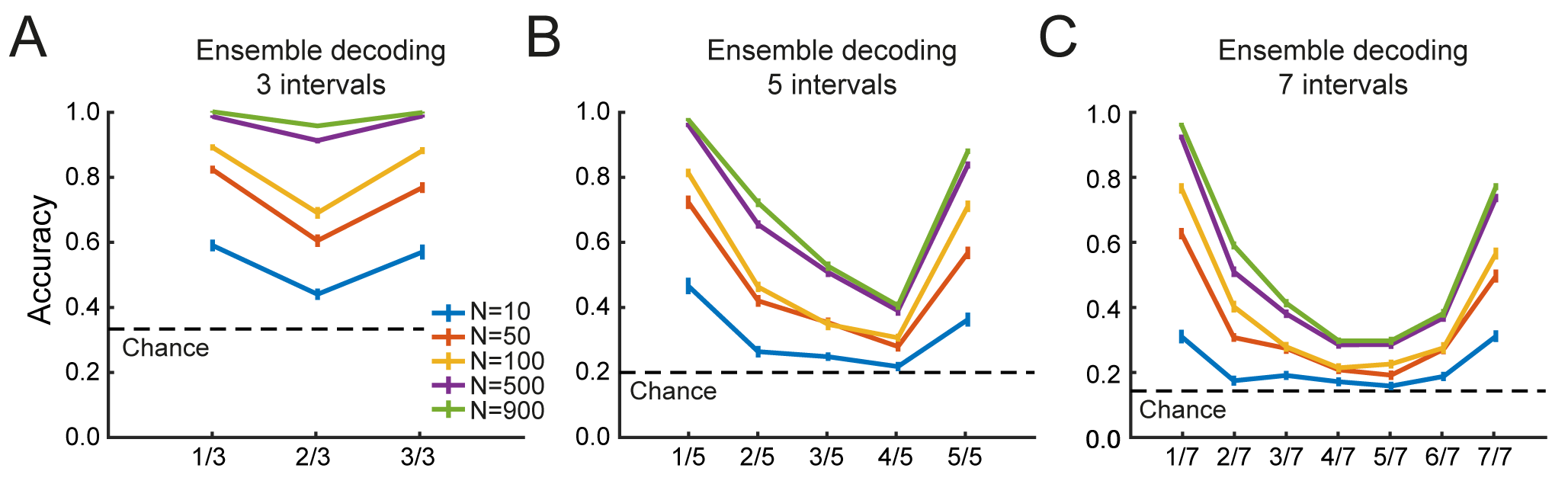

Supplement: Extended Data Figure 3-2 — Decoding analysis with a random forest classifier. A–C, Mean decoding accuracy (±SEM) as a function of pseudo-ensemble size and across time intervals of sequences subdivided in three (A), five (B), and seven (C) equivalently sized consecutive intervals. This figure refers to Figure 3. Download Figure 3-2, TIF file. [file enu-eN-NWR-0279-21-s06.tif]

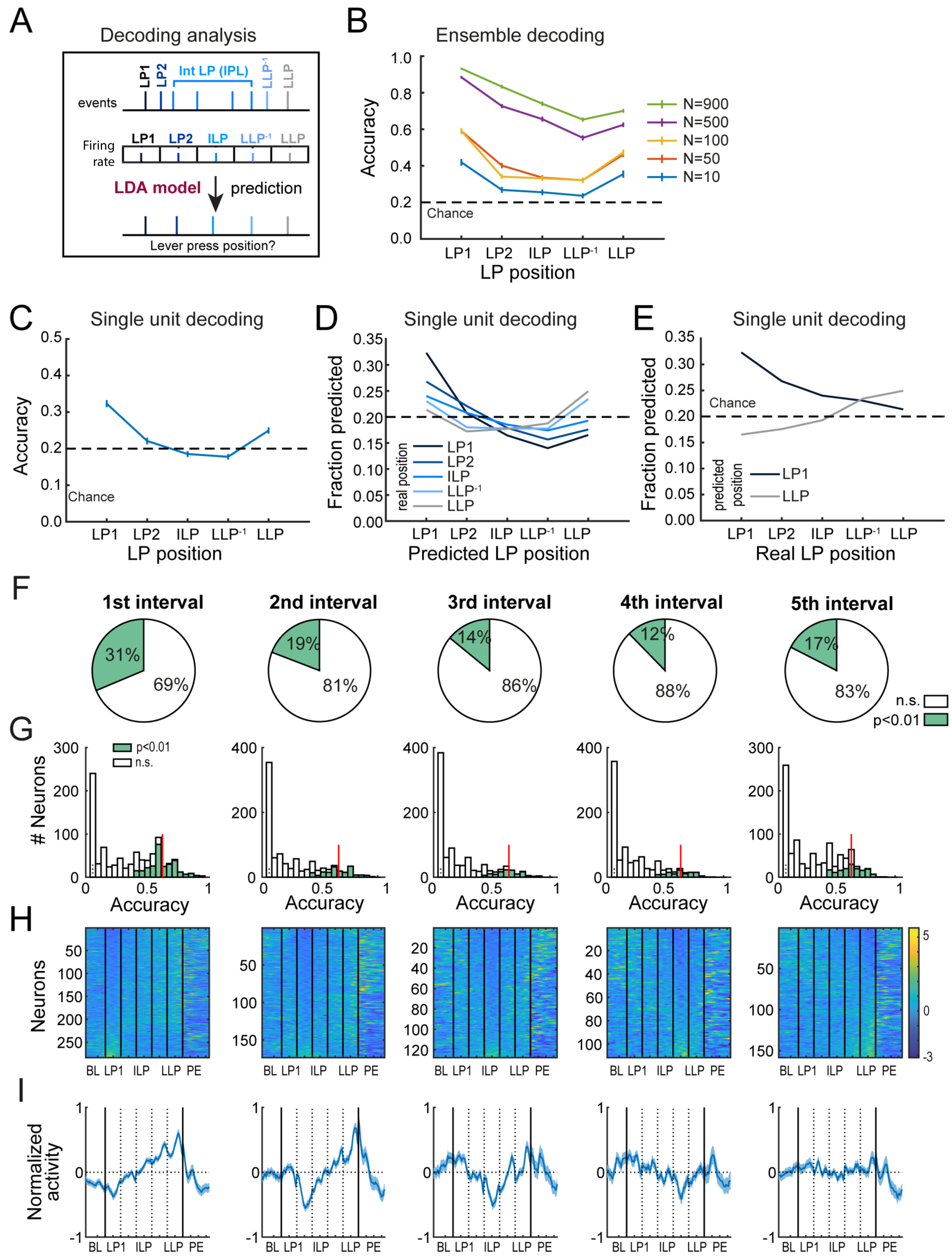

Supplement: Extended Data Figure 3-3 — Progress in the lever press sequence is encoded in DMS activity pattern. A, Diagram of the decoding analysis with an event-centered approach. B, Mean decoding accuracy (±SEM) across lever presses and as a function of pseudo-ensemble size. C, Mean single-unit decoding accuracy (±SEM) across lever presses. D, Fraction of predicted lever press position as a function of real lever press position. E, Fraction of lever press predicted as the first and the last press as a function of real lever press position. F, Proportion of individual neurons that best predicted the position of each lever press event above chance. G, Distribution of decoding accuracy of individual neurons that best predicted each of the lever press events. H, I, Heatmaps (H) and average z score (±SEM; I) of neurons that best predicted the position of a lever press event above chance, for each lever press. This figure refers to Figures 3, 4. Download Figure 3-3, TIF file. [file enu-eN-NWR-0279-21-s07.tif]

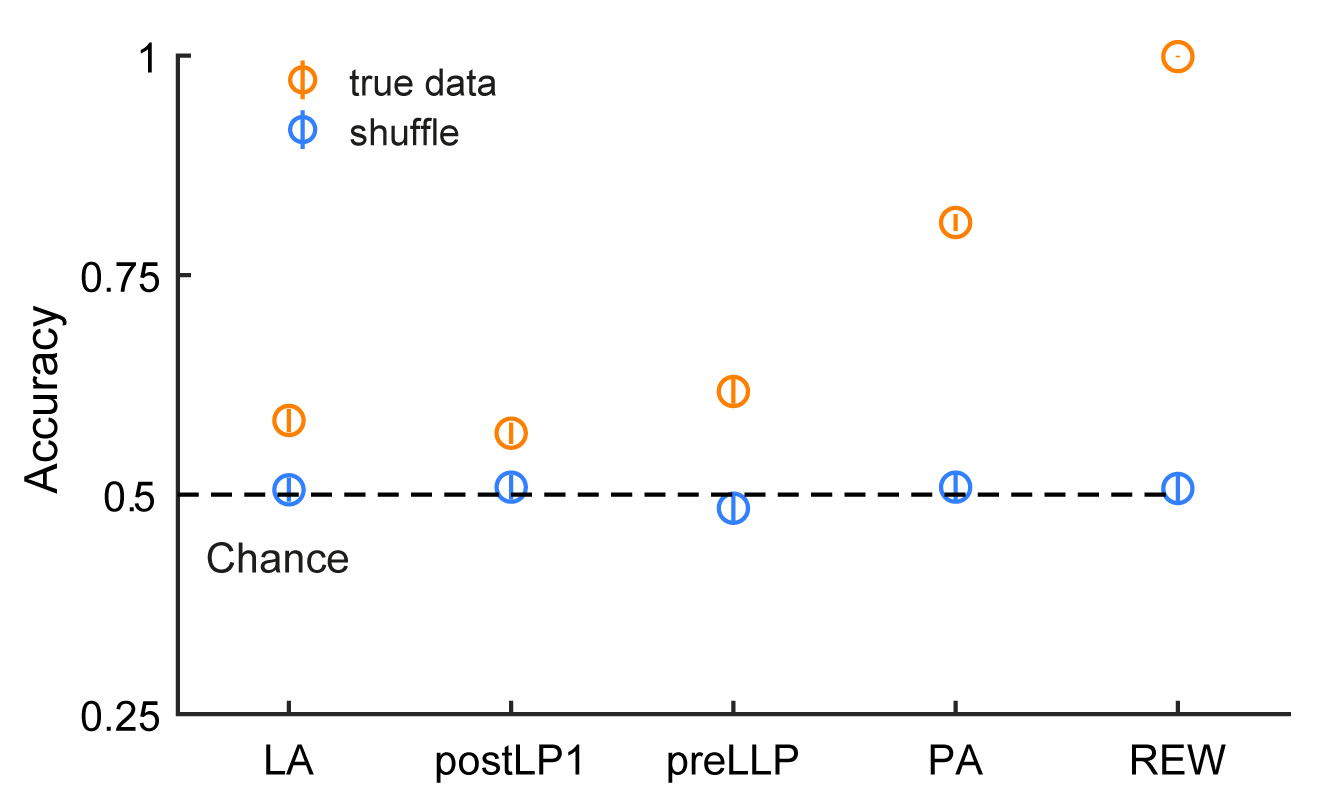

Supplement: Extended Data Figure 5-1 — Decoding of complete versus incomplete sequences with a random forest classifier. Decoding accuracy in true and shuffled conditions across time events in the behavioral sequence. Independent random forest analyses were conducted separately for each time event. This figure refers to Figure 5. Download Figure 5-1, TIF file. [file enu-eN-NWR-0279-21-s08.tif]
